# Pulmonary artery banding in a modified Mustard operation improves biventricular geometry and function

**DOI:** 10.21542/gcsp.2020.36

**Published:** 2020-12-31

**Authors:** Hatem Hosny, Faisal Said, Ahmed Afifi, Walaa Hassan, Mohamed Nagy, Soha Romeih, Magdi Yacoub

**Affiliations:** 1 Aswan Heart Centre, Aswan, Egypt

## Abstract

Patients with transposition of great arteries, with intact interventricular septum (TGA-IVS) and deconditioned left ventricle, represent a considerable challenge in developing countries. The modified Mustard operation was shown to provide a significant improvement for these patients, particularly by enhancing atrial functions and left ventricular filling. Yet, the problems of the systemic right ventricular dysfunction and the resulting secondary tricuspid regurgitation (TR) remain to be of major concern. In addition, the deviation of the interventricular septum towards the left side markedly impairs ventriculo-ventricular interaction and predisposes to dynamic left ventricular outflow tract obstruction (LVOTO). We report that adding a moderately loose pulmonary artery banding to the modified Mustard operation in a case of TGA-IVS results in improvement of biventricular geometry and function, tricuspid and mitral valve functions and disappearance of dynamic LVOTO.

## Introduction

**Figure 1.**
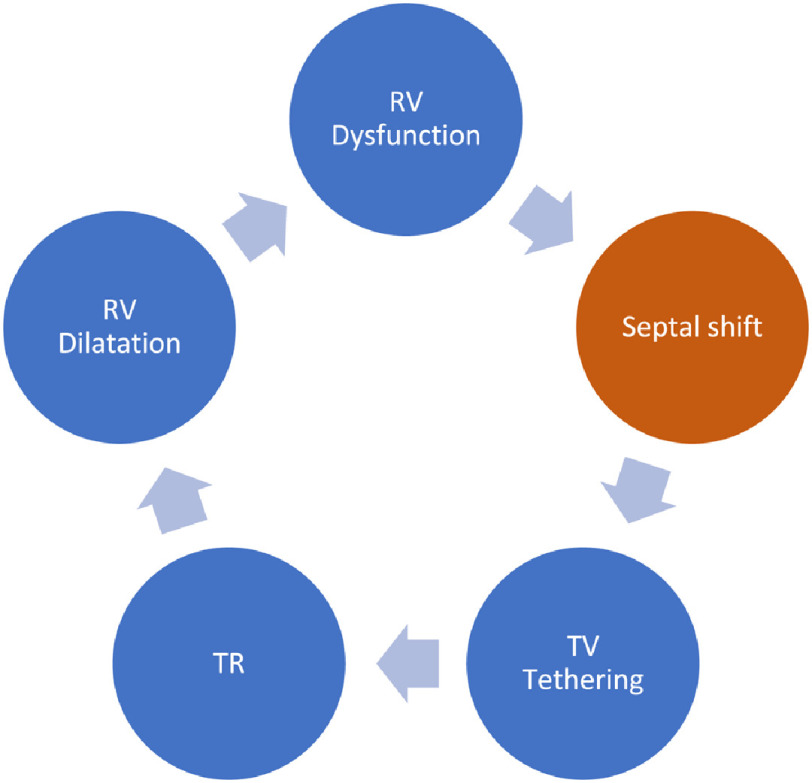
Theory for the establishment of a vicious circle secondary to septal shift.

The modified Mustard operation is being increasingly used for patients with TGA and intact interventricular septum (TGA-IVS) with encouraging results.^1–4^ One of the main objectives of this operation is to improve left ventricular (LV) filling and volume by enhancing the conduit, reservoir and contractile functions of the atrial channels. However, the shift of the IVS is known to increase tricuspid regurgitation due to tethering of the tricuspid septal attachments. The latter can establish a vicious circle producing further dilatation of the right ventricle (RV) and tricuspid regurgitation (TR) (Figure 1).

The shift of the IVS interferes with the physiologic ventriculo-ventricular interaction.^5,6^ In addition, in patients with dynamic left ventricular obstruction, LV filling and volume are hampered by the bulge of the interventricular septum into the LV cavity. We hypothesized that moderately loose banding of the pulmonary artery can interrupt this vicious circle and abolish the dynamic LVOTO. In support of this hypothesis, we here report the immediate hemodynamic effects of loose PA banding during modified Mustard, in a patient with severe TR and dynamic LVOTO.

**Figure 2.**
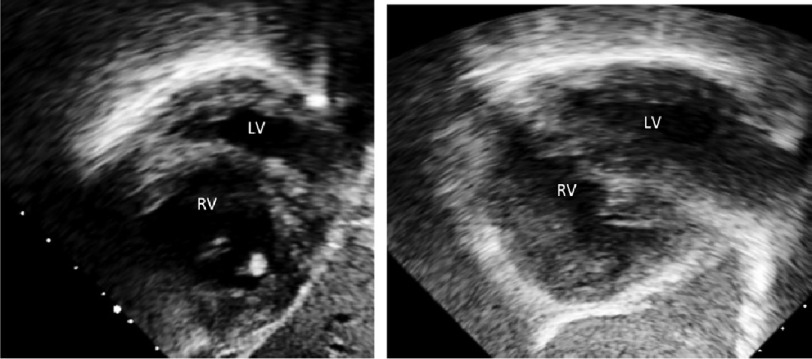
A. Preoperative septal position was deviated towards the left side as shown in trans-thoracic echo (subcostal short-axis view). B. Post-operatively, there was considerable deviation of the septum towards the right side when compared to the pre-operative position.

**Figure 3.**
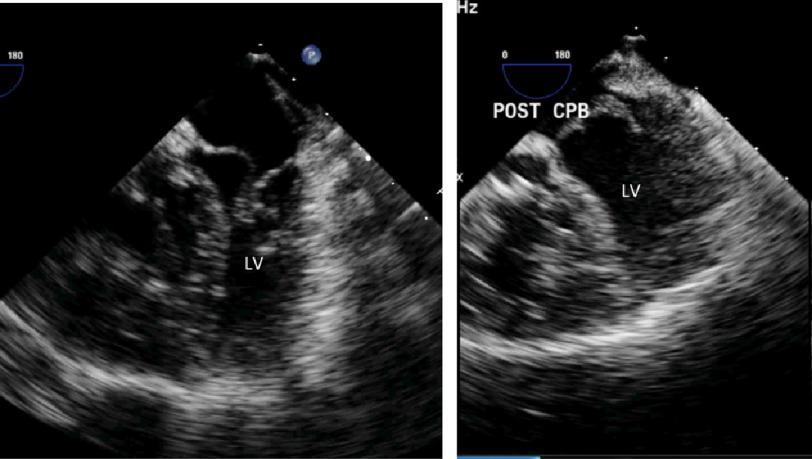
A. Pre-banding “banana-shaped” LV as shown by per-operative trans-esophageal echo. B. Increased LV inflow diameter with “normalization” of LV shape after banding.

**Figure 4.**
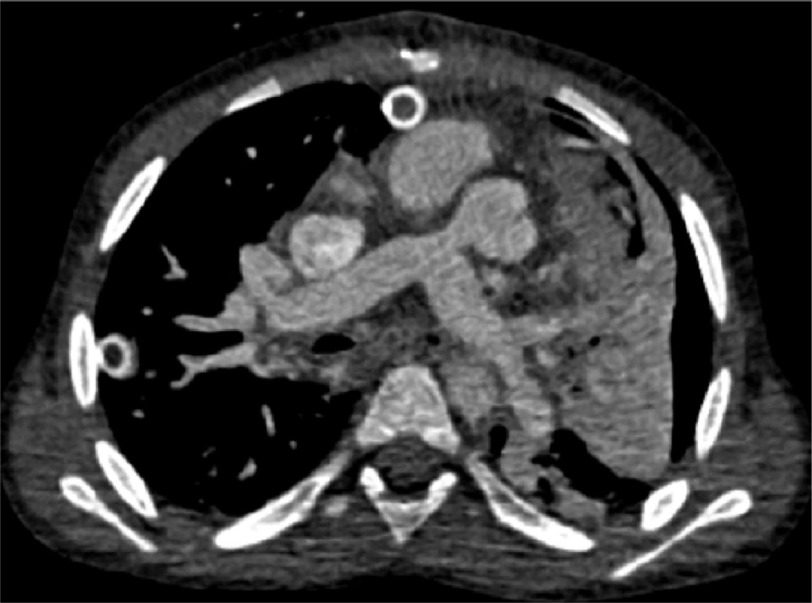
Early post-operative CT image showing the degree of tightness of the pulmonary artery band.

## Patient and Methods

A 6-year-old male patient presented with long-standing cyanosis and clubbing. He had a previous history of balloon atrial septostomy at the age of 5 days. Oxygen saturation was 60% and hemoglobin level was 20.3 gm/dl. Echocardiogram showed complete transposition of the great arteries with intact inter-ventricular septum and adequate-sized ASD. The right ventricle was dilated with moderate impairment of systolic function and severe tricuspid regurgitation (Figure 6A). The IVS was deviated towards the left with systolic anterior motion of the anterior mitral leaflet, causing dynamic LVOTO with peak gradient of 30 mmHg and moderate mitral regurgitation (Figures 6A and 7A).

**Figure 5.**
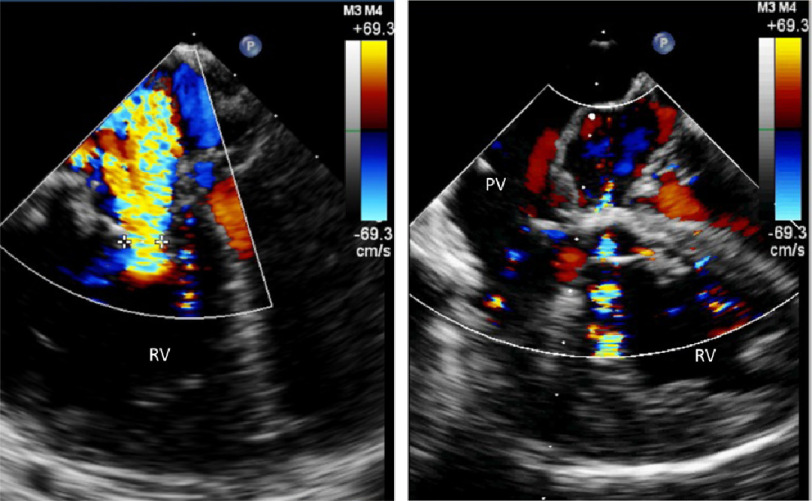
A. Pre-operative color doppler showing severe TR. B. Post-banding image with trivial TR (arrow). RV: Right ventricle, PV: Pulmonary venous channel.

**Figure 6.**
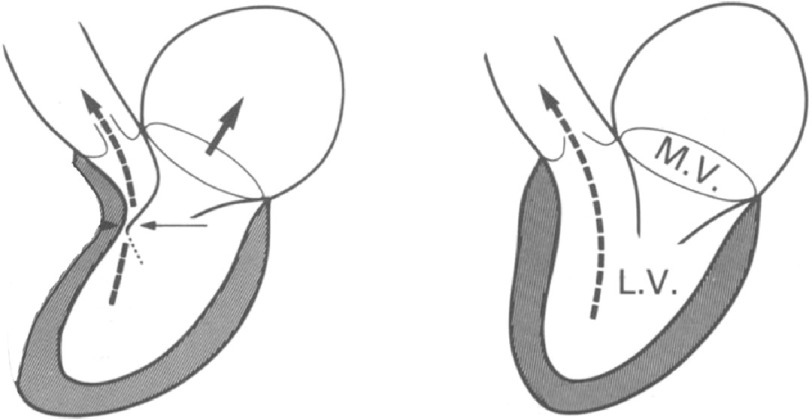
A. Diagram showing the mechanism of mitral regurgitation secondary to systolic anterior motion of the anterior mitral leaflet. B. Post-banding higher LV pressure causes septal deviation and restored competence of the mitral valve.

**Figure 7.**
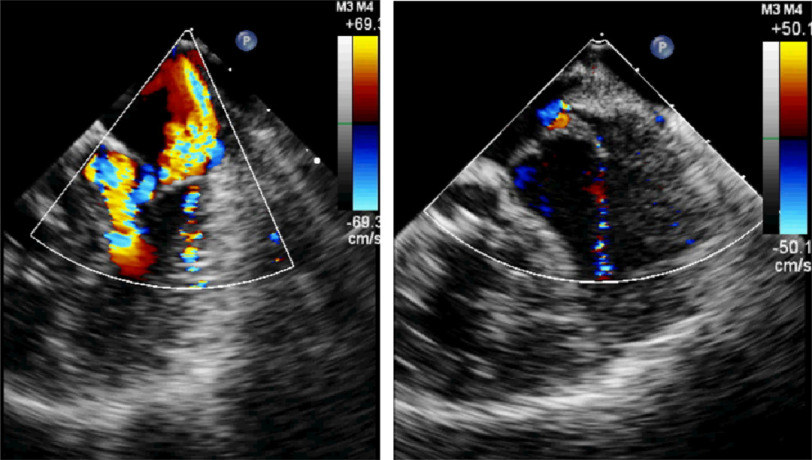
A. Pre-banding color flow mapping echocardiogram showing moderately severe, posteriorly directed mitral regurgitation. B. Post-banding image shows trivial residual mitral regurgitation.

The patient had modified Mustard operation ^1^ and tricuspid valve annuloplasty. After discontinuing cardiopulmonary bypass, a moderately loose pulmonary artery banding was applied under trans-esophageal echo guidance. The systolic pressure proximal to the band (i.e., LV pressure) was 50 mmHg, with systolic aortic pressure of 90 mmHg. The peak gradient across the band was 35 mmHg (Figure 4).

## Hemodynamic, geometric and functional effects of pulmonary artery banding

### A. Position of interventricular septum

Before the operation, the IVS was shifted to the left side. After PAB, the septum was central denoting increased LV pressure (Figure 2).

### B. Left ventricular size and shape

The preoperative banana-shaped left ventricle was transformed to have a much better volume after the operation. The increased volume was associated with preserved ejection fraction (Figure 3).

### C. Right ventricular size and shape

The pre-banding right ventricular globular geometry changed to a “normalized” triangular shape (Figure 2).

### D. Tricuspid valve function

There was severe tricuspid regurgitation pre-operatively. Post-operatively, the TR was trivial. This is the combined effect of the annuloplasty and the decreased tethering due to the effect of the septal shift towards the right (Figure 5).

### E. Mitral valve function

Pre-operatively, there was systolic anterior motion of the anterior mitral leaflet causing moderate mitral regurgitation. This has markedly diminished post-operatively without any surgical intervention to the mitral valve itself. The septal shift abolished the systolic anterior motion (Figures 6 and 7).

### F. Left ventricular outflow tract

**Figure 8.**
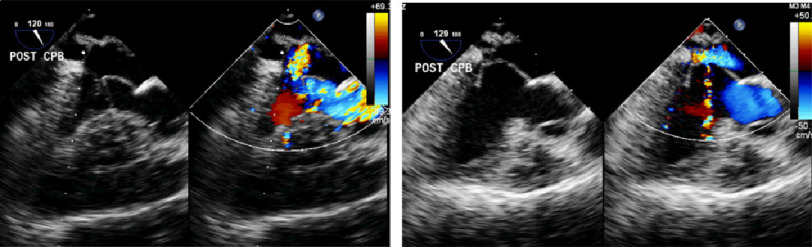
A. Pre-banding echo image showing the dynamic LVOTO with turbulent flow. B. Post-banding image showing wide LVOT with laminar flow.

The dynamic LVOTO caused by the combined septal deviation and the systolic anterior motion virtually disappeared with laminar flow across the LVOT (Figure 8).

## Discussion

Late presenters with TGA-IVS and deconditioned LV continue to represent a major challenge in developing countries.^7^ These patients are classically treated with atrial switch operation which is perceived as an inferior option to the arterial switch operation (ASO) because of the reported continued deterioration in late survival and quality of life.^8^

Trying to improve outcome, we have recently described a modification of the Mustard operation aiming to improve atrial functions and pattern of left ventricular filling.^1^ Yet, the failing systemic RV and significant TR continue to be major problems .^9,10,11^ In an attempt to solve these problems, we have used additional pulmonary artery banding in the patient described in this communication. This was accompanied by significant improvement of geometry and function of both ventricles.

Interestingly, similar changes have been reported when pulmonary artery banding (PAB) was used to re-train the LV in patients who had previous atrial switch or have congenitally corrected TGA (cc-TGA) and presented with failing systemic RV.^12–15^ This was in line with studies reporting the natural history of cc-TGA, showing patients with pulmonary stenosis developed symptoms later, were less handicapped and had a better prognosis than those without pulmonary stenosis.^16^

In conclusion, the findings reported in a single patient strongly suggest that additional PA banding should be considered in patients undergoing the modified Mustard operation, particularly those with impaired RV function, TR or dynamic LVOTO.

## References

[1] Hosny H, Sedky Y, Romeih S, Simry W, Afifi A, Elsawy A (2020). Revival and modification of the Mustard operation. J Thorac Cardiovasc Surg.

[2] Karl TR (2020). Commentary: The Mustard operation is alive and well in Egypt (and elsewhere). J Thorac Cardiovasc Surg.

[3] Chen JM (2020). Commentary: Worldwide, the management of transposition may be more baffling than we knew. J Thorac Cardiovasc Surg.

[4] Bacha E (2020). Commentary: An important contribution to pediatric cardiac surgery in low- and middle-income countries. J Thorac Cardiovasc Surg.

[5] Fogel MA, Weinberg PM, Fellows KE, Hoffman EA (1995). A study in ventricular–ventricular interaction. Circulation.

[6] Yacoub MH (1995). Two hearts that beat as one. Circulation.

[7] Yacoub M, Hosny H, Afifi A (2017). Surgery for TGA in Developing Countries: The End of the Beginning. J Am Coll Cardiol.

[8] Cuypers JaaE, Eindhoven Ja, Slager Ma, Opić P, Utens EMWJ, Helbing Wa (2014). The natural and unnatural history of the Mustard procedure: long-term outcome up to 40 years. Eur Heart J.

[9] Roos-Hesselink JW, Meijboom FJ, Spitaels SEC, van Domburg R, van Rijen EHM, Utens EMWJ (2004). Decline in ventricular function and clinical condition after Mustard repair for transposition of the great arteries (a prospective study of 22-29 years). Eur Heart J.

[10] Duncan BW, Mee RBB (2005). Management of the failing systemic right ventricle. Semin Thorac Cardiovasc Surg.

[11] Daebritz SH, Tiete AR, Sachweh JS, Engelhardt W, von Bernuth G, Messmer BJ (2001). Systemic right ventricular failure after atrial switch operation: midterm results of conversion into an arterial switch. Ann Thorac Surg.

[12] Mavroudis C, Backer CL (2000). Arterial switch after failed atrial baffle procedures for transposition of the great arteries. Ann Thorac Surg.

[13] Padalino MA, Stellin G, Brawn WJ, Fasoli G, Daliento L, Milanesi O (2000). Arterial switch operation after left ventricular retraining in the adult. Ann Thorac Surg.

[14] Winlaw DS, McGuirk SP, Balmer C, Langley SM, Griselli M, Stümper O (2005). Intention-to-treat analysis of pulmonary artery banding in conditions with a morphological right ventricle in the systemic circulation with a view to anatomic biventricular repair. Circulation.

[15] Poirier NC, Mee RBB (2000). Left ventricular reconditioning and anatomical correction for systemic right ventricular dysfunction. Semin Thorac Cardiovasc Surg Pediatr Card Surg Annu.

[16] Bjarke BB, Kidd BS (1976). Congenitally corrected transposition of the great arteries. A clinical study of 101 cases. Acta Paediatr Scand.

